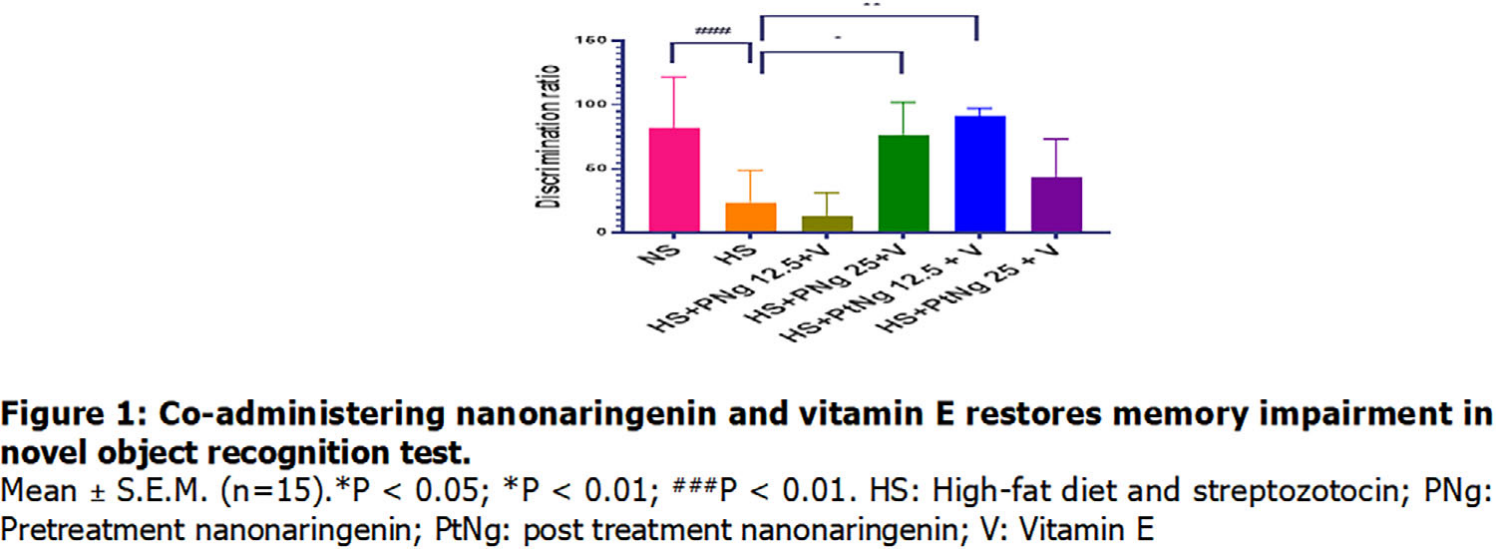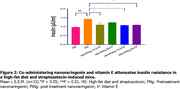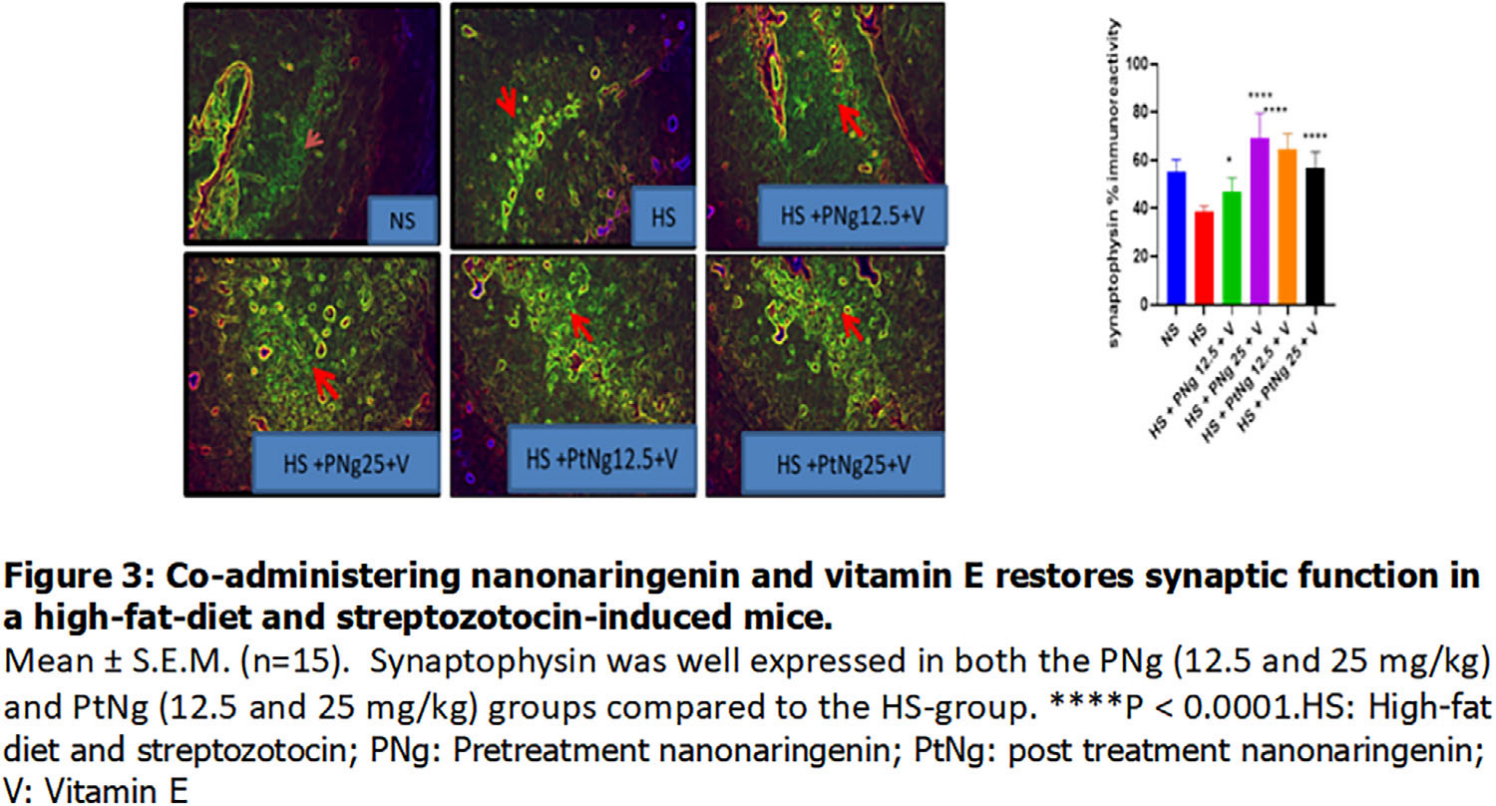# Coadministering nanonaringenin and vitamin E enhances hippocampal memory function and synaptic transmission in diabetic mice

**DOI:** 10.1002/alz.085254

**Published:** 2025-01-09

**Authors:** JULIET NNENDA OLAYINKA, Gabriella Egede, Victoria Madu, Hilda Alari, Chiamaka Ezenwobi, Cherechi Ekeh, Anita Dimm Igochukwu, Oluwole Abidemi Akawa, Abiola Oluwatosin Obisesan

**Affiliations:** ^1^ AFE BABALOLA UNIVERSITY, ADO‐EKITI Nigeria; ^2^ UNIVERSITY OF BENIN, BENIN CITY Nigeria; ^3^ Afe Babalola University, Ado‐Ekiti, Ekiti State Nigeria; ^4^ Afe‐Babalola University, Ado‐Ekiti, Ado‐Ekiti, Ekiti State Nigeria; ^5^ Afe‐Babalola University, Ado‐Ekiti, Ekiti State Nigeria

## Abstract

**Background:**

Diabetic conditions are associated with alterations in brain functions like memory deficits through processes like synaptic dysfunction in the hippocampus. Administering a combination of silver nanonaringenin and vitamin E appears promising since they are known to prevent diabetes and memory deficits in previous studies, and nanoformulation of naringenin may be one way to improve delivery and bioavailability of naringenin in the brain. This study investigated the effects of co‐administering silver nanonaringenin and vitamin E against memory deficits and synaptic dysfunction in the hippocampus of a mice model of high‐fat diet and streptozotocin (HS).

**Method:**

Ninety male mice (n = 15) were divided into six groups and fed with high‐fat diet except the normal group for 28 days. Diabetes was induced by streptozotocin (50 mg/kg intraperitoneally) on day 22. Weight and blood glucose levels were taken every week. On day 15, mice in the pretreatment groups (PNg 12.5 + Vit E 200 mg/kg and PNg 25 + Vit E) started treatment while on day 29, mice in the posttreatment groups (PtNg 12.5 + Vit E and PtNg 25 + Vit E) commenced treatment till day 42. Memory function (Y‐maze and novel object recognition test (NORT)) were performed. The hippocampal cornus ammonus 1 (CA1) was harvested for biochemical assays using ELISA (acetylcholinesterase enzyme (AChE) and insulin test). Immunofluorescence was used in detecting the expression of synaptophysin in the brain.

**Result:**

Nanonaringenin and vitamin E restored memory function, and significantly decreased weight, glucose, insulin, and AChE levels in the HS‐treated mice compared to the HS group. Synaptophysin was scarcely expressed in the pretreatment (12.5 mg/kg) but densely expressed in the Pretreatment 25 mg/kg and Posttreatment (12.5 and 25 mg/kg) groups compared to the HS‐group.

**Conclusion:**

Co‐administering silver nanonaringenin and vitamin E restored hippocampal memory function through reversing insulin resistance, cholinergic mechanism and restoring synaptic function in the CA1 of the hippocampus of HS‐induced mice.